# Design, Preparation, and Bioactivity Study of New Fusion Protein HB-NC4 in the Treatment of Osteoarthritis

**DOI:** 10.3389/fbioe.2021.700064

**Published:** 2021-08-18

**Authors:** Yaya Wang, Lian Li, Qiang Wei, Rongrong Chai, Qingqiang Yao, Chen Liang, Fuwen Wang, Yan Li

**Affiliations:** ^1^ Institute of Materia Medica, Shandong First Medical University & Shandong Academy of Medical Sciences, Jinan, China; ^2^ Key Laboratory of Chemical Biology (Ministry of Education), School of Pharmaceutical Sciences, Cheeloo College of Medicine, Shandong University, Jinan, China; ^3^ Department of Physical Education, Tangshan Normal University, Tangshan, China; ^4^ Shandong Medical College, Jinan, China

**Keywords:** osteoarthritis, C5b-9, HB, NC4, fusion protein

## Abstract

Osteoarthritis (OA) is now becoming the main disease that affects public health. There is no specific medicine used for OA in clinical application until now. Recently, several studies demonstrated that OA is closely related to the complement system, and some complement regulators such as N-terminal non-collagenous domain 4 (NC4) aimed at alleviating OA have shown a promising therapeutic effect. However, targeting ability is the main limitation for NC4. In this study, a fusion protein named heparin-binding domain-N-terminal non-collagenous domain 4 (HB-NC4) was proposed to solve this problem, which could provide a better way for OA treatment. First, HB-NC4 plasmid was constructed using ClonExpress II one-step ligation kit method. And *Escherichia coli* BL21 was utilized to express the fusion protein, Ni^2+^-sepharose, and a desalting gravity column were introduced to purify HB-NC4. The results showed that 0.84 mg HB-NC4 could be obtained from a 1 L culture medium with a purity higher than 92.6%. Then, the hemolytic assay was introduced to validate the anti-complement activity of HB-NC4; these results demonstrated that both HB-NC4 and NC4 had a similar anti-complement activity, which indicated that heparin-binding (HB) did not affect the NC4 structure. Targeting ability was investigated *in vivo*. HB-NC4 showed a higher affinity to cartilage tissue than NC4, which could prolong the retention time in cartilage. Finally, the destabilization of the medial meniscus (DMM) model was applied to investigate HB-NC4 pharmacodynamics *in vivo*. The results indicated that HB-NC4 significantly slowed cartilage degradation during the OA process. In summary, compared with NC4, HB-NC4 had better-targeting ability which could improve its therapeutic effect and prolonged its action time. It could be used as a new complement regulator for the treatment of OA in the future.

## Introduction

**FIGURE 1:**
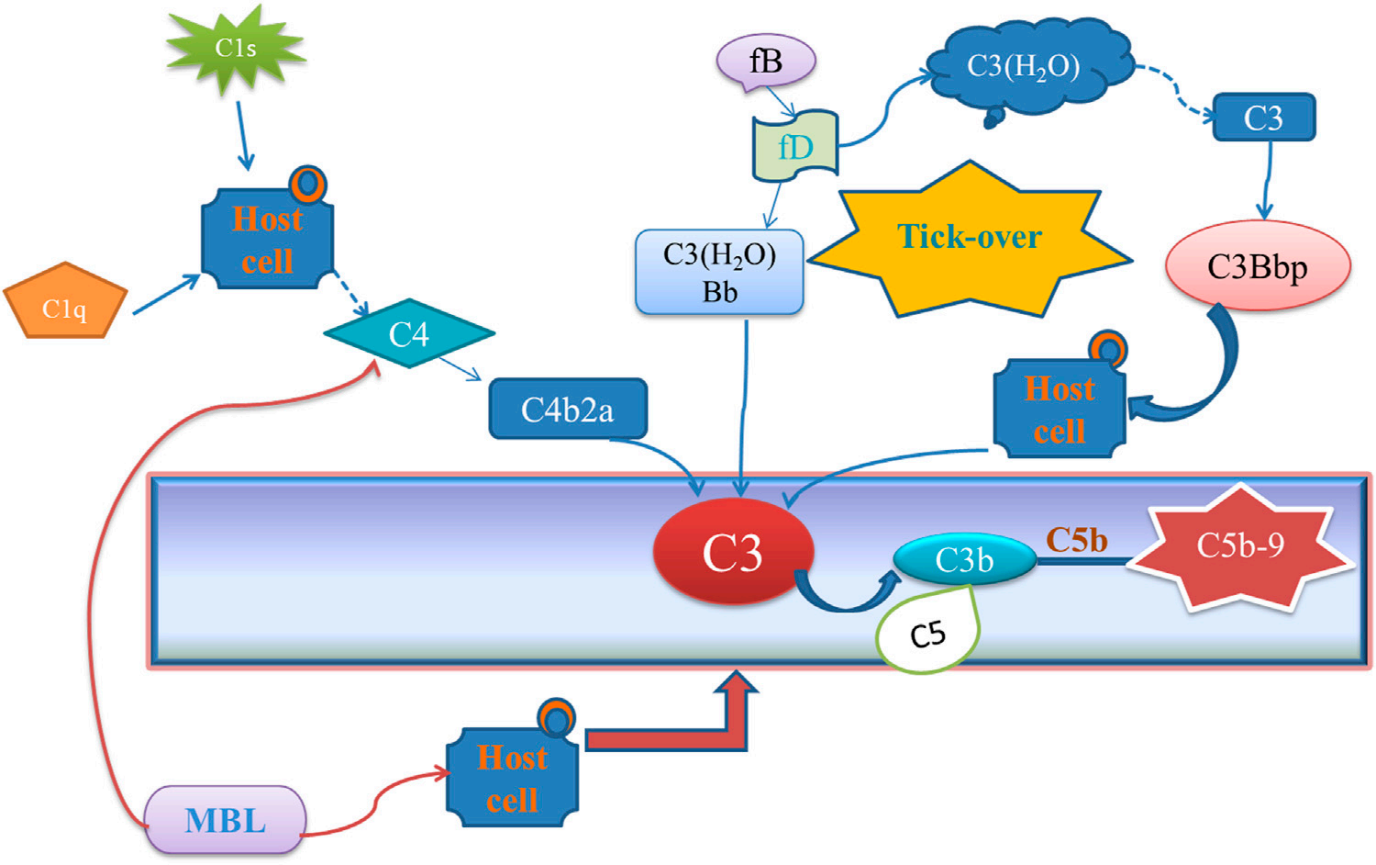
Different complement activation pathways.

Osteoarthritis (OA) is a kind of degenerative disease that is mainly caused by joint trauma, chronic injury, or overuse of the knee. Accumulating evidence indicates that OA is mainly caused by low-grade inflammation (Robinson et al., 2016). Complement system activation plays a key role during the low-grade inflammatory process of OA (Wang et al., 2011; Struglics et al., 2016). A complement system is a multi-molecular system consisting of a group of proteins that can be provided with enzymatic activity after activation in the serum and tissue fluid of normal humans and animals. However, excessive activation of the complement system will trigger an immune response and promote inflammation (Melis et al., 2015). The complement system is activated *via* classical pathway, alternative pathway, or lectin pathway, which can generate complement component C3, eventually formed C5b-9 (Figure 1). Complement activation final product, membrane attack complex (MAC, C5b-9), can form on the cell and directly cause the chondrocyte death by osmotic flux (Heinen et al., 2009). Also, MAC can promote the production of matrix metalloproteinases (MMPs) and cyclooxygenase which induces the formation of OA (Matrai et al., 2019). Therefore, how to block the complement activation, especially MAC, will become a potential strategy for OA treatment (Evans, 2005; Fortier et al., 2011). Recently, several anti-inflammatory protein inhibitors have shown promising potentials in alleviating OA on animal models level (Ru and Robert, 2015; Michelfelder et al., 2018), such as insulin-like growth factor 1 (IGF-1) and the *N*-terminal non-collagenous domain 4 (NC4) (Schmidt et al., 2006; Kalchishkova et al., 2011), which could directly inhibit the classical pathway of complement. NC4 is part of cartilage collagen IX and could pass through the fibril surface and provide interaction sites for other matrix components (Sjöberg et al., 2007). Loss of the NC4 domain is the early event in cartilage degradation in joint diseases (Pihlajamaa et al., 2004; Carlsen et al., 2006). Some research demonstrates that NC4 could be a complement inhibitor to relieve the inflammation in OA by preventing C9 polymerization, C3, C4 activation, and lower the MAC level finally (Blom et al., 2003; Kalchishkova et al., 2011). Normally, NC4 is applied through intra-articular injection (Danfelter et al., 2007). However, as a protein drug, the retention time and targeting ability should be improved. Therefore, how to make therapeutic proteins target cartilage with a longer retention time through intra-articular injection is the key to the clinical application (Farach-Carson et al., 2005; Evans et al., 2011).

A novel fusion protein HB-NC4 was described here, which could combine targeting ability and therapeutic effect (Loffredo et al., 2014). The HB domain could make the fusion protein have better-targeting ability and longer existing time in the extracellular matrix (Farach-Carson et al., 2005; Chu et al., 2013) while NC4 could display its strong complement inhibitory activity *via* regulating complement activity (Kalchishkova et al., 2011; Brown et al., 2020). Through this research, we would like to provide a new strategy for OA treatment.

## Materials and Methods

### Cloning and Expression of Recombinant Protein HB-NC4 in *E. coli*

The sequence of the HB-NC4 gene was designed according to the *E. coli* codon usage preference. The pET-HB-NC4 and pET-NC4 were obtained by cloning the HB-NC4 and NC4 genes into the pET28a (+) (6 × His tag) vector between the *BamH* I/*Xho* I restriction sites using the ClonExpress II one-step cloning kit (Vazyme Biotech, China) (Sun et al., 1993; Li et al., 2010). The expression clone was transformed into *E. coli* BL21 (DE3) (Tiangen, China). The obtained recombinant plasmid HB-NC4 and NC4 were confirmed by colony PCR and DNA sequencing analysis. Single colonies picked from the LB solid medium plate were grown in LB medium containing kanamycin sulfate (100 µg/ml) at 37°C and 200 rpm on an orbital shaker for 2–3 h to an OD_600_ of 0.6–0.8. Isopropyl-β-D-thiogalactoside (Sigma, China) was added to a final concentration of 0.25 mM under different conditions, including 25°C for 16 h, 37°C for 4 h, and 37°C for 6 h. The wet cells pellet was centrifuged at 8,000 × g at 4°C and suspended in equilibration buffer (20 mM PB, 500 mM NaCl, 5 mM imidazole, pH 7.4). The cells were sonicated on ice using a sonicator for 30 min at output 30% with repeated 3 s on/5 s off. The HB-NC4 and NC4 in a soluble fraction and pellet fraction were analyzed by SDS-PAGE.

### Purification of Fusion Proteins

Ni^2+^-sepharose (GE Healthcare) affinity chromatography was used to purify the NC4 and HB-NC4. The impurity proteins were eluted with Ni^2+^-sepharose elution buffer (imidazole, 50 mmol/L), and the target protein was collected by Ni^2+^-sepharose collection buffer (imidazole, 250 mmol/L). The collected proteins were condensed with the ultrafiltration tube and desalted using a gravity desalination column, acquiring HB-NC4 and NC4 proteins with higher purity. SDS-PAGE and Western blot were used to identify the fusion proteins HB-NC4 and NC4, and ImageJ (1.8.0) was used for purity analysis.

### Western Blot Analysis

The fusion proteins were verified by Western blot analysis using rabbit source anti-body with 6 × His-tag. The fusion proteins were resolved on an SDS-PAGE gel (15%) and then transferred onto PVDF membrane in transfer buffer (Tris base 5.82 g, glycine 2.93 g, SDS 0.375 g, water 800 ml, and methanol 200 ml) for 30 min at 15 V. Following the transfer, the membranes were blocked in 5% nonfat milk (nonfat milk 5 g, TBST 100 ml) for 1 h. The membrane was incubated overnight at 4°C with a specific primary antibody of 6 × His-tag. The membrane was then applied to a secondary antibody conjugated to goat anti-rabbit for 1 h. Finally, the membrane was analyzed by Azure Biosystems C400.

### Hemolytic Assay

Sheep erythrocytes were used for assays of the classical pathway of complement, and rabbit erythrocytes were used for the alternative pathway (Dijk et al., 1980; Seelen et al., 2005; Hong et al., 2017; Zhang et al., 2020). We study alternative pathway with rabbit erythrocytes, which were washed three times with the Mg^2+^EGTA buffer (veronal 0.26 g, NaCl 2.1 g, MgCl_2_ 0.71 g, EGTA 1.9 g, glucose 12.6 g, and gelatin 0.5 g, added water to 500 ml, PH 7.4). The concentration of the rabbit erythrocytes suspension was adjusted by deionized water until the absorbance at 405 nm up to 1 ± 0.2. The 7% serum and increasing concentrations of test proteins (20, 40, 60, 80, 100 μg/ml) were added to a 96-well plate for 15 min on ice. Blank group (without serum and test protein) and control group (without test protein) were set up at the same time. Then, about 10 μL adjusted suspension was added at once; the subsequent reaction was at 37°C for 1 h. Plates were centrifuged after the incubation, and the lysis of erythrocytes was measured at 405 nm using the microplate reader (BioTek SYNERGY H1).

Sheep erythrocytes were washed three times with ice-cold DGVB^2+^ buffer (2.5 mM veronal buffer, pH 7.4, 70 mM NaCl, 1 mM MgCl_2_, 0.15 mM CaCl_2_, 140 mM glucose, and 0.1% gelatin) and incubated with the same volume complement-fixing antibody for 20 min at 37°C. Erythrocytes were then washed twice in ice-cold DGVB^2+^ buffer and incubated in a 96-well plate together with 0.7% serum and increasing concentrations (20, 40, 60, 80, 100, 120 μg/ml) of test protein for 1 h at 37°C. Blank group (without serum and test protein) and control group (without test protein) were set up at the same time. Plates were centrifuged after the incubation, and the lysis of erythrocytes was measured at 405 nm using a microplate reader.

### Affinity Assay

The immunohistochemical assay was performed using the anti-His antibody in tissue sections from mice articular cartilage at the given times after intra-articular injection of NC4, HB-NC4, or PBS. C57BL/6 mice (as shown in Table 1) were purchased from Jinan Pengyue Experimental Animal Breeding Co., Ltd. The sex of mice does not influence the results of the study, but the same experimental period is best for the same sex. By injecting HB-NC4 (80 μg/kg), NC4 (80 μg/kg), or PBS alone into the knee joints of adult mice, after 6 h, we took out the knee joints cartilage tissue, invaded into paraformaldehyde 4%. Immunohistochemistry needed to be processed, including decalcification, dehydration, paraffin embedding, sectioning, and histochemical staining, and tested whether fusion with a heparin-binding domain altered the kinetics of retention in joint tissues.

**TABLE 1:** The design of animal experiment used for both the affinity study and pharmacodynamics of HB-NC4 and NC4.

Groups		Treatments	Time of sacrifice
Affinity study	Control group (n = 3)	Intra-articular injection with PBS	6 h
	HB-NC4 group (n = 3)	Intra-articular injection with 80 μg/kg HB-NC4	6 h
	NC4 group (n = 3)	Intra-articular injection with 80 μg/kg NC4	6 h
Pharmacodynamics study	Control group (n = 8)	Intra-articular injection with PBS per week	12 weeks
	Model group (n = 8)	Intra-articular injection with PBS per week	12 weeks
	HB-NC4 high dosage group (n = 8)	Intra-articular injection with 60 μg/kg per week	12 weeks
	HB-NC4 low dosage group (n = 8)	Intra-articular injection with 15 μg/kg per week	12 weeks
	NC4 high dosage group (n = 8)	Intra-articular injection with 60 μg/kg per week	12 weeks
	NC4 low dosage group (n = 8)	Intra-articular injection with 15 μg/kg per week	12 weeks

### DMM-Model for Therapy of OA

Eight-week-old male C57BL/6 mice (Jinan Pengyue Experimental Animal Breeding Co., Ltd.) were used in this study. Mice were kept in the animal breeding room at 21 ± 2°C with 40 ± 15% relative humidity under relatively clean circumstances, housed four per cage, and provided with food and water. All animals care and experiments were in accordance with the National Institute of Health Guide for the Care and Use of Laboratory Animals and approved by the Ethics Committee of Institute of Materia Medica Shandong Academy of Medical Science (Approval no. 201905, Jinan, Shandong Province, China).

Mice were acclimatized for a week before the experiment. Mice were divided into six groups (as shown in Table 1), five groups were induced to OA models. All mice are anesthetized by injecting chloral hydrate (10%) into the abdominal cavity. The DMM was established as previously described (Miller et al., 2014; Jeong et al., 2018). In short, the skin on the medial of the right knee joint was cut about 1 cm. The medial meniscus tibial ligament was exposed by blunt dissection and transected. The five OA model mice were randomly assigned to one of five treatment groups: weekly intra-articular injection of 60 μg/kg HB-NC4, 15 μg/kg HB-NC4, 60 μg/kg NC4, 15 μg/kg NC4, or PBS only into the right knee joint, the remaining group that was only cut a skin incision as a sham group, which was injected with PBS once a week. The intra-articular injection was implemented on the second day after surgical transection of the medial meniscus tibial ligament and lasted for 12 weeks (7 days/week). Blood in the orbital sinus of all mice was collected, centrifuged to obtain serum, and kept at −80°C for ELISA; subsequently, all mice were sacrificed (Zhao et al., 2014). The right knee joints were collected and fixed in 4% paraformaldehyde and then used for safranin O-fast green staining and immunohistochemical analyses.

### ELISA Analysis

The collected serum from every mouse was unfrozen. All reagents in the ELISA kit were placed at room temperature for 15–30 min. The tested serum of every experimental mouse was diluted in a ratio of 1: 5 with sample diluent. The serum and standards (7.5 μg/l, 15 μg/l, 30 μg/l, 60 μg/l, 120 μg/l) were added into microplates and incubated at 37°C for 30 min. After pouring out the liquid and washing the plates, the microplates were added anti-C5b-9 conjugated to HRP for 30 min. After the reaction solution was discarded, chromogenic solutions were added and then stopped after 10 min with stop solution. The absorbance was measured using a microplate reader (BioTek SYNERGY H1) at 405 nm.

### Safranin O-Fast Green Staining

The keen joints were taken out from 4% paraformaldehyde, washed several times with water to remove paraformaldehyde, and then soaked in 10% EDTA solution to decalcify for 3 weeks. The decalcification solution was replaced every 2 d. The joint tissue was dehydrated with gradient alcohol and made transparent with a transparent agent (xylene). The transparent tissue block was soaked in paraffin wax, which passed through three cylinders of paraffin (60°C). Tissue blocks soaked in wax were embedded at slightly higher than 60°C. Paraffin-embedded tissue sections of articular cartilage were deparaffinized to accomplish safranin O-fast green staining. For identifying the nuclei, Harris hematoxylin was applied for 2 min. The beginning staining was conducted with a fast green stain for 3 min and then soaked in 1% glacial acetic acid for 3 min. Subsequently, the tissue sections were soaked in safranin O stain for 3 min (Armin et al., 2020). Stained tissues were dehydrated with absolute ethanol and transparent with xylene and subsequently mounted on slides using neutral balsam. Images of each slide were obtained with a biological microscope (Olympus BX53, Japan). The results of safranin O-fast green staining are treated by scoring the degree of cartilage damage (Harden et al., 2013).

### Immunohistochemical Staining

The reserved paraffin-embedded tissue sections of articular cartilage were deparaffinized to conduct immunohistochemical analyses. The sections were preincubated in 1% trypsin solution for antigen retrieval. The tissues were blocked with 5% BSA in PBS for 1 h. A specific primary antibody against C5b-9 was incubated overnight with the sectioned tissues (15 h). Subsequently, the tissue sections were incubated for 1 h with HRP-labeled goat anti-rabbit IgG or anti-mouse IgG. The location of the antigen in the tissue was determined with diaminobenzidine (DAB) for 10 min, and the nuclei were identified with Harris hematoxylin for 2 min. Stained tissues were dehydrated and subsequently mounted on slides using neutral balsam. Images of each slide were obtained with a biological microscope (Olympus BX53, Japan).

### Statistical Analysis

The results were expressed as mean ± SEM for each treatment group in each experiment. Statistical analyses were performed using the statistical product and service solutions (22.0). Statistical differences between two groups were analyzed by two-tailed Welch’s *t*-test and a non-parametric one-way ANOVA followed by Tukey’s multiple comparison’s test was performed on three or more groups of data.

## Results and Discussions

### Construction of Recombinant Plasmid pET28a(+)-HB-NC4 and pET28a(+)-NC4

**FIGURE 2:**
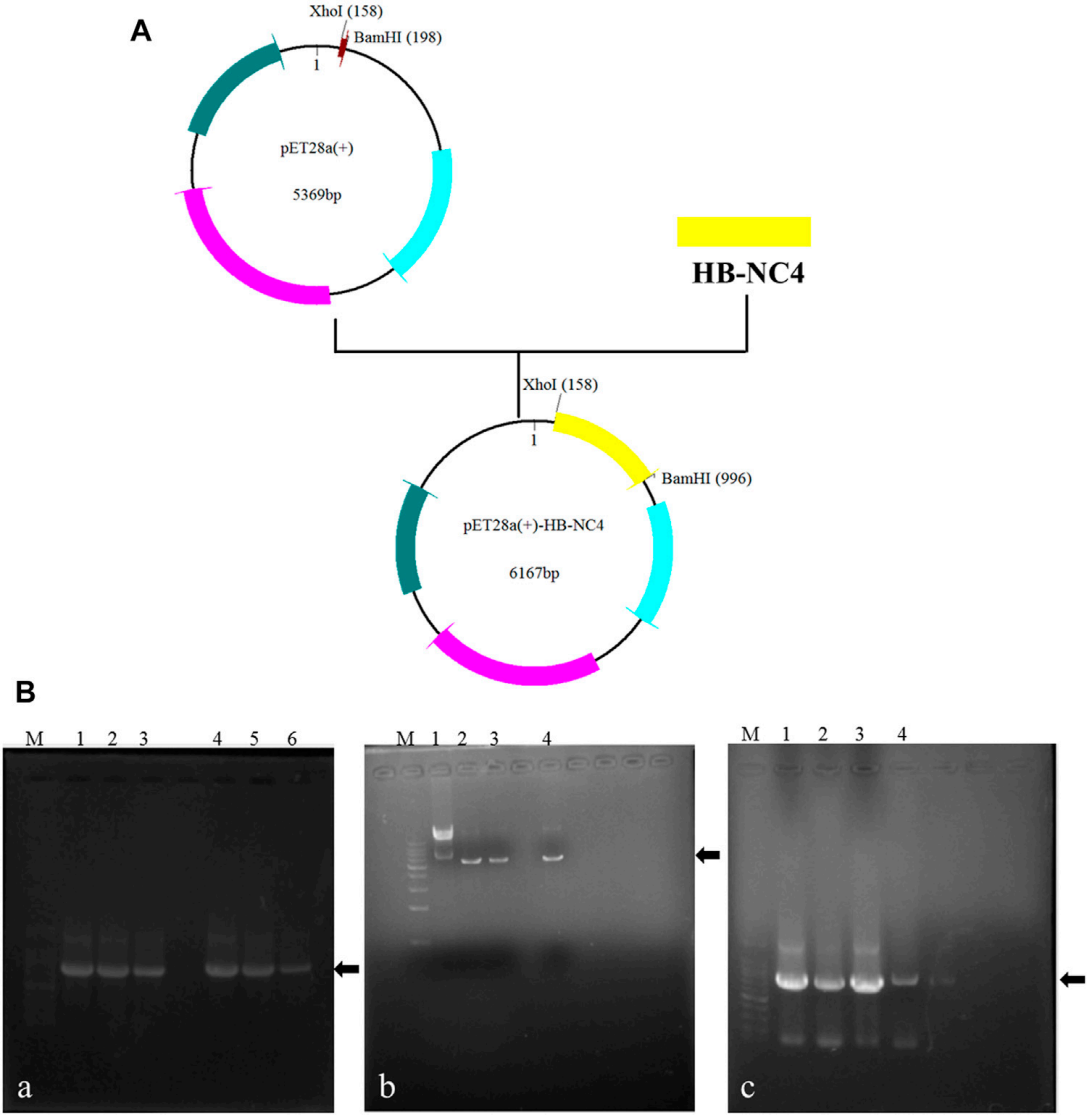
Construction of recombinant plasmids **(A)**; recombinant plasmids of pET28a (+)-NC4 and pET28a (+)-HB-NC4. The results of the construction of recombinant plasmids pET28a (+)-HB-NC4 and pET28a (+)-NC4 **(B)**, **(A)** Agarose gel electrophoresis (AGE) of amplification products of target genes by PCR. Lanes 1–3 are NC4 and lanes 4–6 are HB-NC4. **(B)** The results of double restriction digestion of plasmid. Lane 1 is pET28a (+) empty plasmid. Lanes 2–4 are plasmid digestion products. **(C)** The positive transformants were screened by PCR. Lanes 1–3 are NC4. Lane 4 is HB-NC4.

In order to increase the targeting ability and retention time of NC4, HB was fused to the *N*-terminal (Kalchishkova et al., 2011). The structures of recombinant plasmids are shown in Figure 2A. The results of PCR amplification of target genes are shown in Figure 2BA; each PCR product was of the expected size. PCR verification of the colonies obtained is shown in Figure 2BC. The obtained plasmids were sequenced prior to further study and each yielded the correct sequences.

### Expression and Purification of HB-NC4 and NC4

**FIGURE 3:**
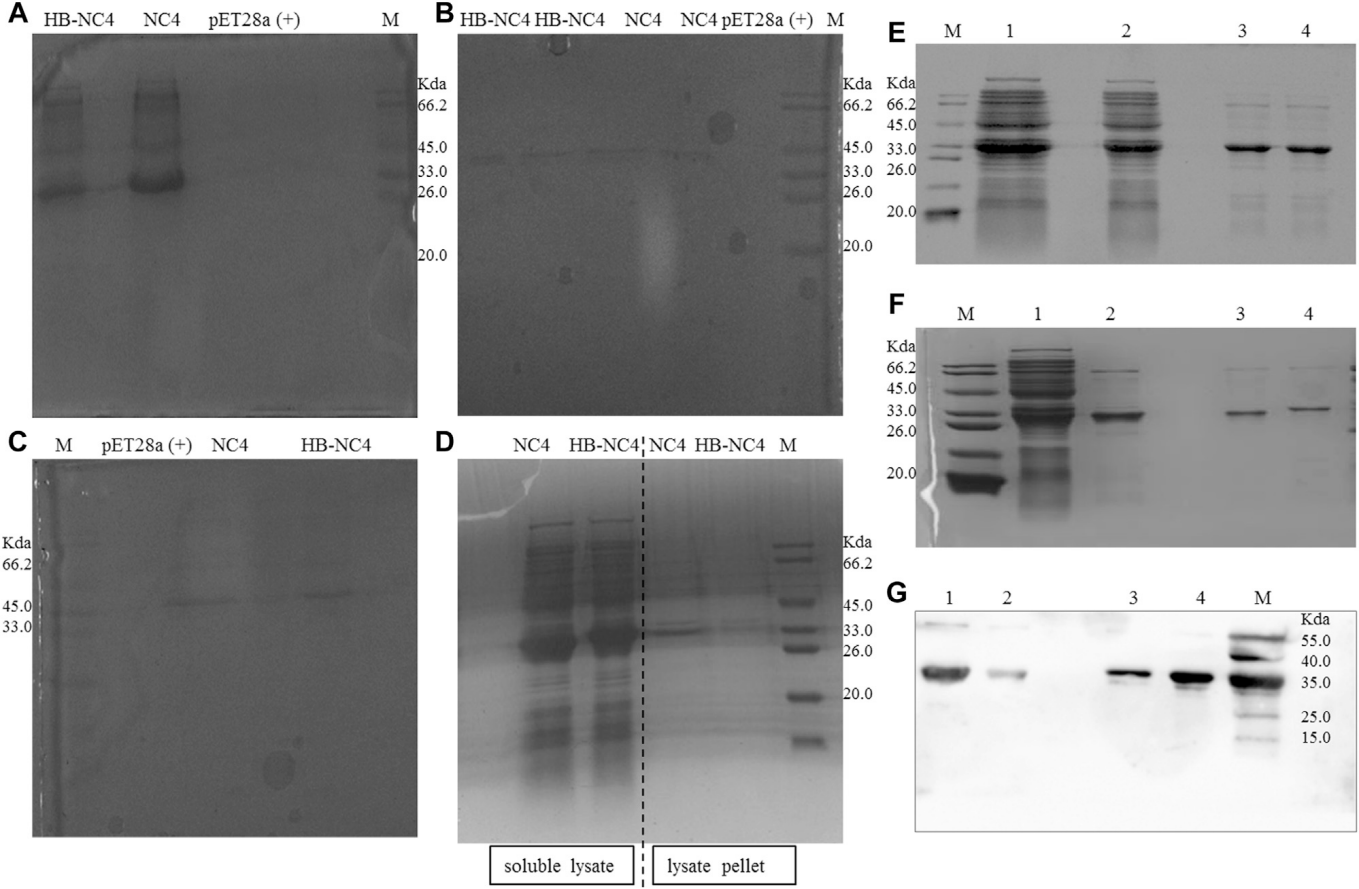
SDS-PAGE analysis of the expression of HB-NC4 and NC4 **(A–D)**. The soluble lysate of pET28a (+), NC4, and HB-NC4 at 37°C for 6 h **(A)**. The expression in the lysate pellet of NC4 and HB-NC4 at 37°C for 6 h **(B)**. The soluble lysate of NC4 and HB-NC4 at 37°C for 4 h **(C)**. The soluble lysate of NC4 and HB-NC4 at 25°C for 16 h **(D)**. The molecular weight of the recombinant protein HB-NC4 predicted by the gene sequence is approximately 32.6 kDa. Purification result of HB-NC4 **(E)**; lanes 1 and 2 are the unpurified fractions while lanes 3 and 4 represent the purified HB-NC4 by Ni^2+^-sepharose. Purification result of fusion protein NC4 **(F)**; lane 1 is the unpurified fractions, lane 2 is that NC4 was purified by Ni^2+^-sepharose, and lane 3 is desalination of NC4. Lane 4 is the desalination of HB-NC4. Western blot analysis of the purification of the proteins **(G)**. Lane 1 is that HB-NC4 was purified by Ni^2+^-sepharose, lane 2 is desalination of HB-NC4, lane 3 is desalination of NC4, and lane 4 is that NC4 was purified by Ni^2+^-sepharose.

Different expression conditions for HB-NC4 and NC4 were investigated and the results are shown in Figure 3. From Figures 3A–C, it could be found that when the temperature was 37°C, there was almost no protein expressed in both the soluble form and inclusion body. Therefore, a lower temperature fermentation method was introduced and the results are shown in Figure 3D. The results indicated that HB-NC4 and NC4 could be successfully expressed in the soluble form at 25°C for 16 h. During the fermentation process, temperature and fermentation time was investigated (Davis et al., 2015). We found that low temperature could increase the expression of HB-NC4 in soluble form, while high temperature perturbed the expression of HB-NC4. Therefore, 25°C was selected as the optimized parameter. Since the temperature was confirmed, it was necessary to discuss the fermentation time. Usually, when the temperature was low, it needs more time to get the fusion protein. Inducer was very important to protein expression. Our previous work found that IPTG concentration had little influence on the expression of HB-NC4. Therefore, the concentration of IPTG was not optimized in this study (Pan et al., 2008).

Purification was another key procedure for HB-NC4. In this study, the 6 × His tag was introduced to help purify the fusion protein from the supernatant. HB-NC4 and NC4 were purified by Ni^2+^-sepharose affinity chromatography and SDS-PAGE results (shown in Figures 3E,F) indicated that the purity of both proteins was higher than 90.8 and 92.7%, respectively. Further, fractions containing HB-NC4 were pooled and dialyzed against phosphate-buffered saline (PBS) to desalt; however, protein precipitation occurs. Finally, both proteins were desalted by gravity desalting column, which could affect the following *in vitro* and *in vivo* experiments (Ren et al., 2014; Peng et al., 2015). 0.84 mg HB-NC4 could be obtained from 1 L culture media with purity at approximately 92.6%, and 1.12 mg NC4 was acquired from 1 L culture media at approximately 94.1% purity. Western blot was applied to validate the product, and it could be concluded that HB-NC4 and NC4 were successfully obtained.

Fusion proteins could also be acquired from inclusion bodies. But protein refolding should be overcome (Michelfelder et al., 2018). Protein refolding occurred easily in inclusion bodies which needs a complex process including release, isolation, washing, denaturation, and renaturation (Zhang et al., 2015). Based on the expression of HB-NC4 under different experimental conditions, the yield in the supernatant was significantly higher than that in the inclusion bodies (Lilie et al., 1998; Hartinger et al., 2010), and the supernatant purification strategy was selected. Finally, the Western blot results indicated that fusion proteins were successfully expressed and purified in this study.

### Anti-Complement Activity of HB-NC4 *In Vitro*

**FIGURE 4:**
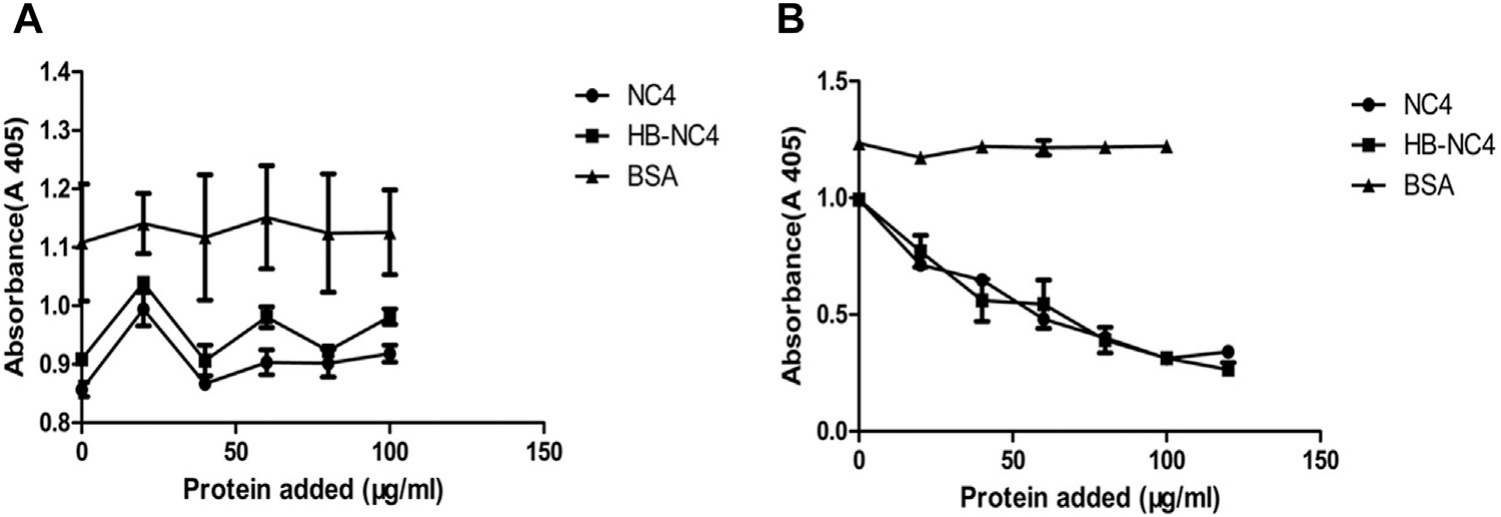
Inhibition of alternative pathway-mediated lysis of erythrocytes by NC4 and HB-NC4 **(A)**. Rabbit erythrocytes were used for the alternative pathway-mediated lysis, respectively, and BSA was used as a negative control. Sheep erythrocytes were used for the classical pathway-mediated lysis, respectively, and BSA was used as negative control **(B)**. The graphs represent mean values from three independent experiments performed in duplicate ± S.D.

The inhibiting complement activity of HB-NC4 and NC4 was evaluated by hemolytic assays (Amin et al., 1997). The anti-complement activities of HB-NC4 and NC4 for the classical pathway and alternative pathway were investigated. As shown in Figure 4A, it could be found that both proteins showed no inhibitory effect to the alternative pathway and the results were in accordance with Kalchishkova’s results (Kalchishkova et al., 2011). However, a significant inhibitory effect on the classical pathway (Figure 4B) could be observed, which was in accordance with our hypothesis (Sandy et al., 1984; Fang and Beier, 2014). The IC_50_ values of HB-NC4 and NC4 were 38 μg/ml and 42 μg/ml, indicating that HB had no effect on the anti-complement activity of NC4 and had a certain enhancement effect which may be caused by the fact that HB could interact with the glycosaminoglycans in the cartilage. Also, the inhibitory effect was dose-dependent, and when the concentration of HB-NC4 was increased to 80 μg/ml, the inhibition rate could reach 80%.

### The Affinity of HB-NC4 and NC4 to Cartilage Tissue

**FIGURE 5:**
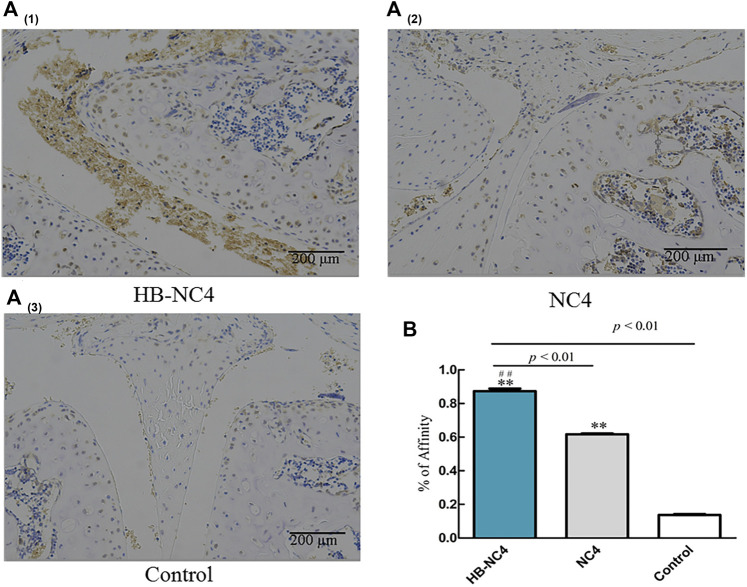
The affinity of HB-NC4 to cartilage tissue **(A)**. After analyzing, we found that HB-NC4 can remain in the joint cartilage tissue without being removed. Results are summarized in bar graphs showing inhibition rate (cross) of different protein drugs (line) in OA mice joints **(B)**. The mean ± SEM, for HB-NC4, 0.93 ± 0.06, for NC4, 0.61 ± 0.16, and for control, 0.15 ± 0.06 (n = 3 per group). ^##^ *p* < 0.01, **p < 0.01.

We investigated the affinity of HB-NC4 to cartilage using immunohistochemistry methods. And the results in Figure 5A showed that, in the same concentration and time, there were more HB-NC4 specifically retained in both articular and meniscal cartilage than NC4, since HB could interact with glycosaminoglycans in cartilage. The statistics results showed that the affinity of HB-NC4 (80 μg/kg) group and NC4 (80 μg/kg) group to cartilage tissue was significantly higher than that of the control group, and the affinity of HB-NC4 group to cartilage tissue was significantly higher than that of NC4 group (Figure 5B). Thus, HB could strongly improve the pharmacodynamic effect of NC4 (Fang and Beier, 2014).

### Pharmacodynamics of HB-NC4 and NC4 on DMM-Induced OA

**FIGURE 6:**
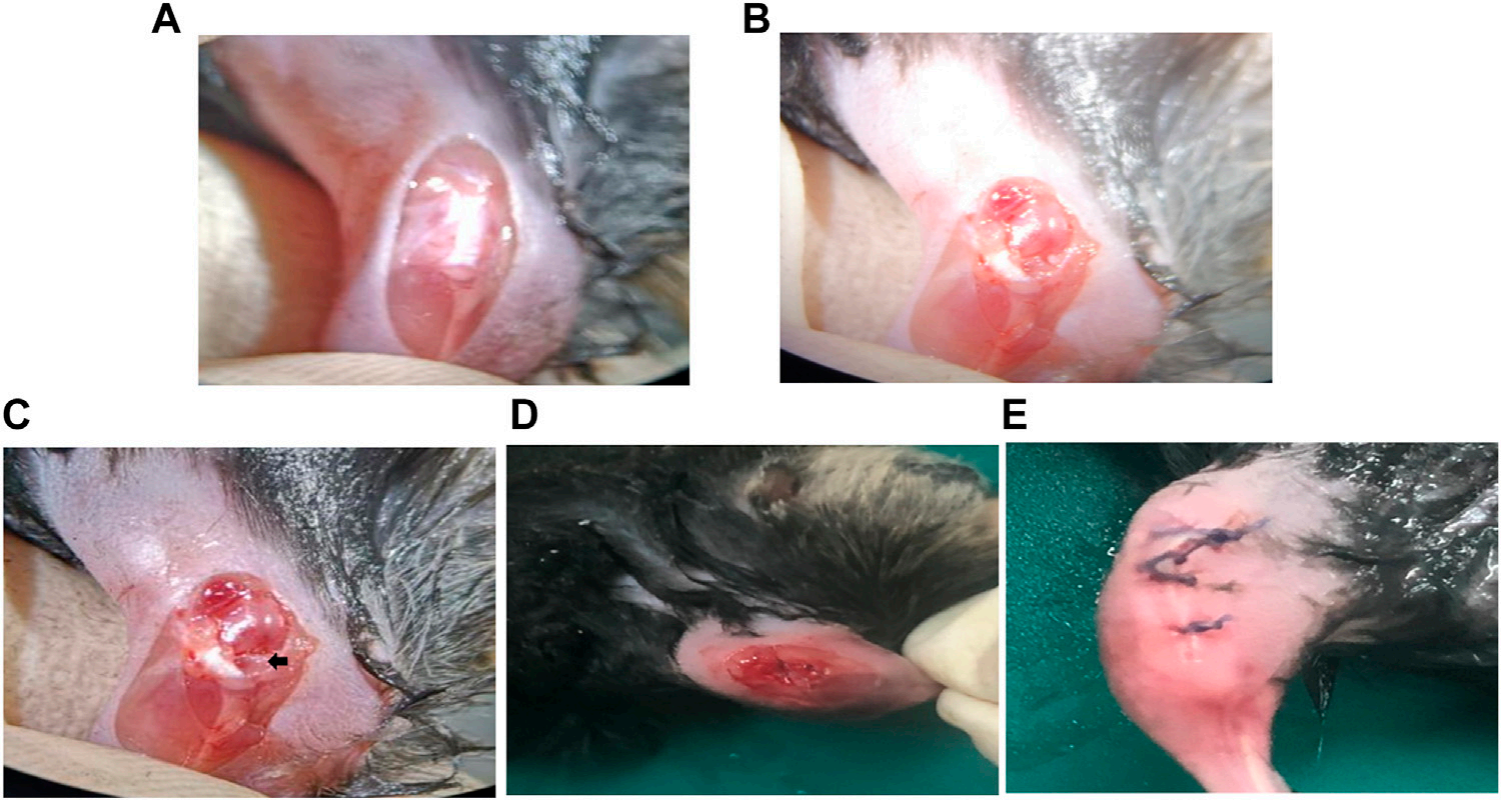
Surgery steps of DMM model establishment. The joint capsule was exposed **(A)**. The medial meniscus tibial ligament was exposed. **(B)**. The tibial ligament was transected **(C)**. The joint capsule is sutured **(D)**. The knee joint of the mouse after surgery **(E)**.

The establishment of the OA model is greatly essential. Currently, the OA model commonly includes drug inducing model, obesity inducing model, gene inducing model, and a surgical-inducing model (Lim et al., 2015). In this study, a mouse destabilization of the medial meniscus (DMM) was selected to simulate mild or moderate OA. The DMM model was successfully established by cutting the tibial ligament of the medial meniscus of the mice (Figures 6A–E). The advantage of this method is that the model is convenient and can simulate OA caused by damaged joints of humans, but the disadvantage is that it is not suitable for simulating more serious OA (Miller et al., 2010; Zaki and Little, 2018). Whether the model was successfully established could be identified by observing the cartilage damage of the model group after staining with safranin O-fast green as shown in Figure 8 (Jeong et al., 2018). HB-NC4, NC4, or PBS was injected into the knee joints once a week for 12 weeks. Then, all mice were sacrificed for ELISA, histologic, and immunohistochemical analysis.

**FIGURE 7:**
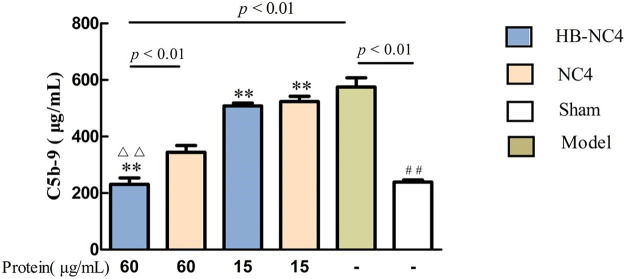
The effect of HB-NC4 and NC4 on the content of C5b-9 in mouse serum. The bar graphs (mean ± SEM, n = 8) represent quantitative results of the C5b-9 obtained from a microplate reader. ^##^ *p* < 0.01, ^△△^ *p* < 0.01, **p < 0.01.

The ELISA results showed that compared with the sham group, the serum C5b-9 content in the model group was significantly higher, indicating that the model was successful (Figure 7). Compared with the model group, the serum C5b-9 content in the HB-NC4 low-dose and HB-NC4 high-dose groups and NC4 low-dose and NC4 high-dose groups were significantly reduced (**p < 0.01). Compared with the NC4 high-dose group, the serum C5b-9 content in the HB-NC4 high-dose group was significantly lower (^△△^ *p* < 0.01), which demonstrated that HB-NC4 showed better effects.

**FIGURE 8:**
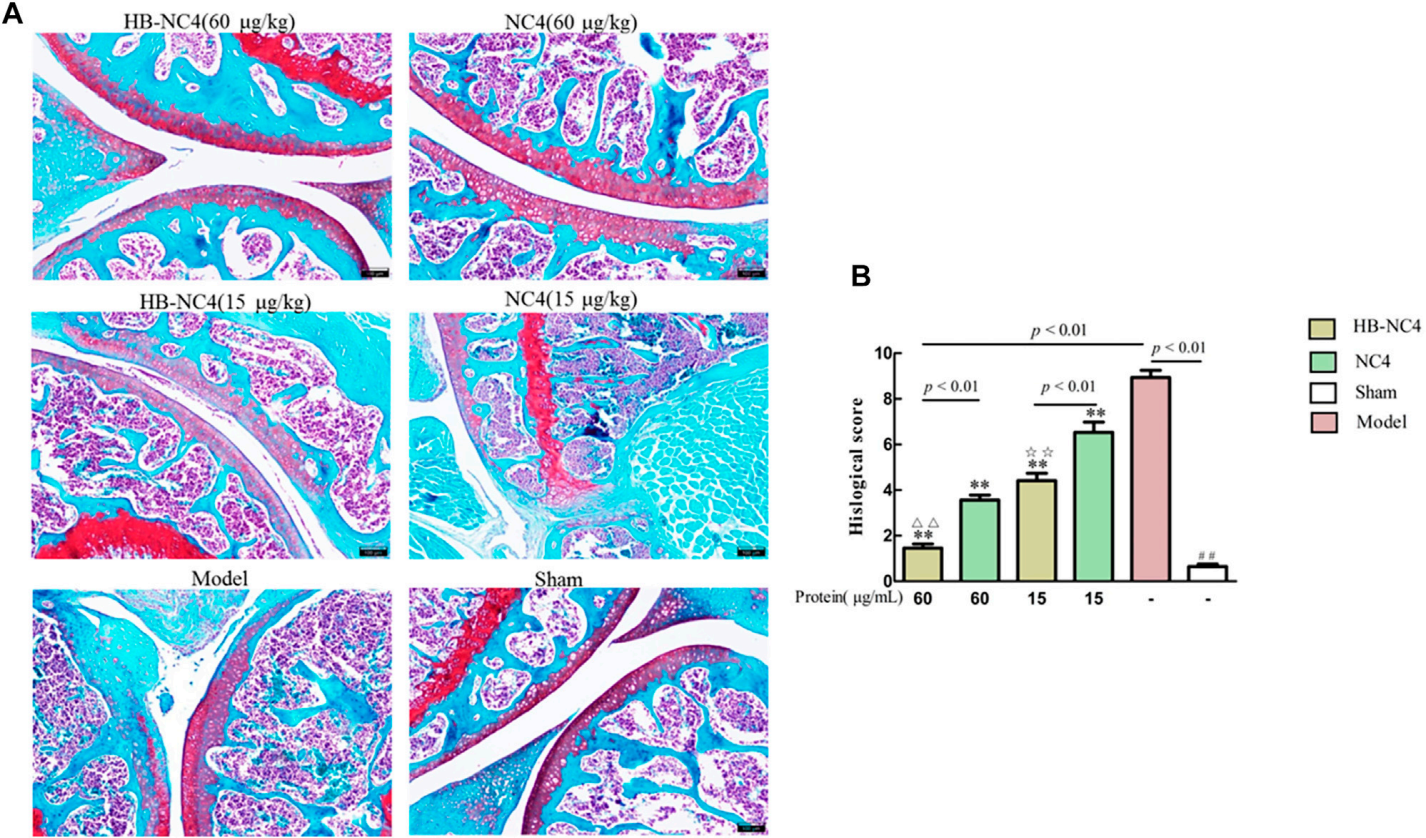
afranin O-fast green staining of the joint cartilage **(A)**. Results are summarized in bar graphs (n = 8) showing the therapeutic effect of different protein drugs (line) on cartilage damage degree (cross) **(B)**. ^##^ *p* < 0.01, ^△△^ *p* < 0.01, ^☆☆^ *p* < 0.01, **p < 0.01.

The results of safranin O-fast green staining showed that there was little cartilage loss in the sham group, and cartilage loss in the model group was greater than 50%, indicating that the DMM-induced OA model was successfully established (Figure 8A). Compared with the model group, HB-NC4 low-dose group, HB-NC4 high-dose group, and NC4 low-dose and NC4 high-dose groups were significantly different, all had different degrees of cartilage loss, and all had significant differences (Figure 8B). Compared with NC4 low-dose and NC4 high-dose groups, respectively, HB-NC4 low-dose and HB-NC4 high-dose groups could significantly reduce the degree of cartilage damage in animals (^☆☆^ *p* < 0.01 or ^△△^ *p* < 0.01).

**FIGURE 9:**
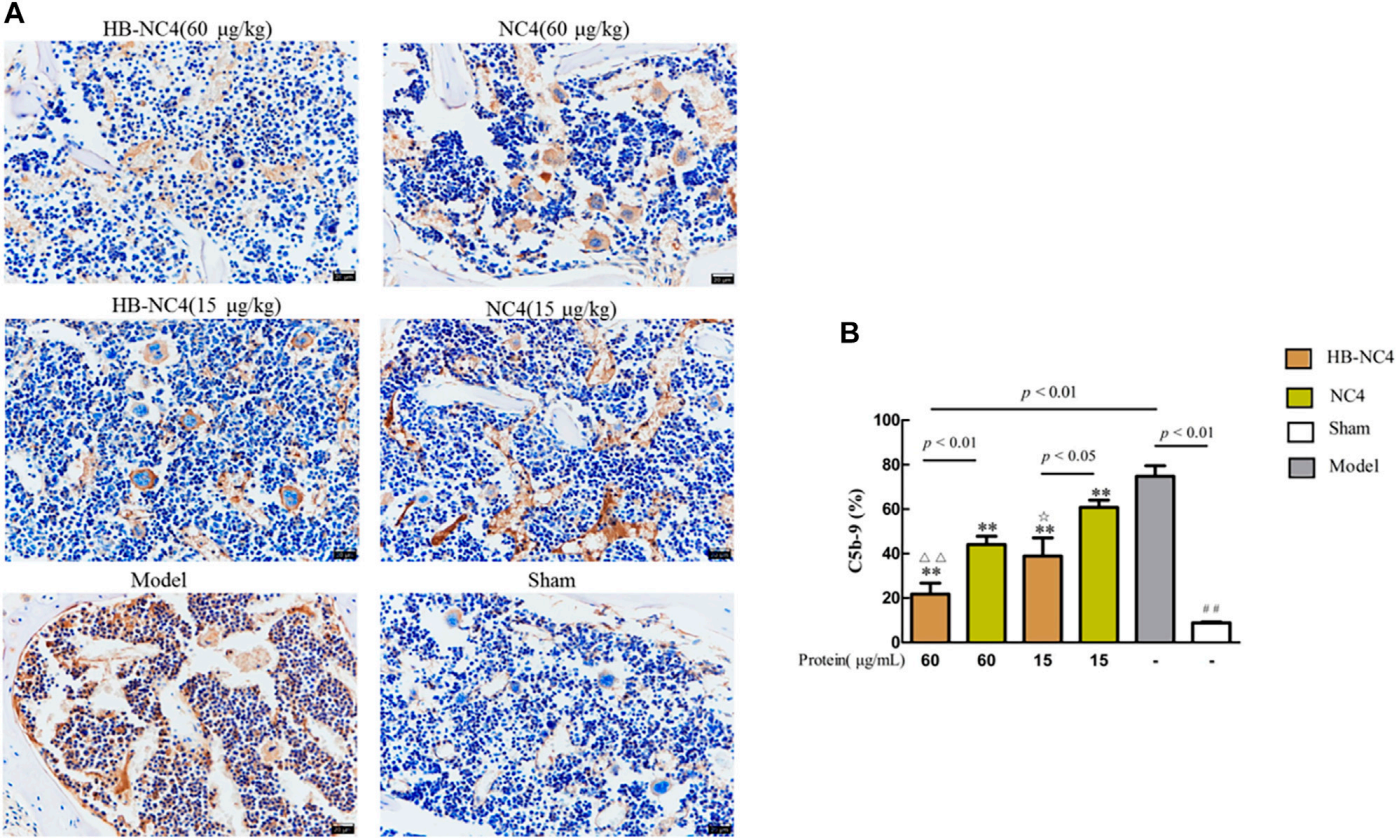
Analysis results of C5b-9 content in cartilage tissue **(A)**. Results are summarized in bar graphs (n = 8) showing inhibition rate (cross) of different protein drugs (line) on C5b-9 generation in OA mice joints **(B)**. Compared with sham group (^##^ *p* < 0.01), compared with NC4 (60 μg/kg) group (^△△^ *p* < 0.01), compared with NC4 (15 μg/kg) group (^☆^ *p* < 0.05), and compared with model group (**p < 0.01).

The results of immunohistochemistry showed that the content of C5b-9 in the model group was significantly higher (^##^ *p* < 0.01) (Figure 9A). Compared with the model group, the distribution of C5b-9 on the surface of articular cartilage of mice in HB-NC4 low-dose group, HB-NC4 high-dose group, and NC4 low-dose and NC4 high-dose groups was significantly decreased (Figure 9B). Compared with NC4 low-dose and NC4 high-dose groups, respectively, the distribution of C5b-9 on the surface of articular cartilage of mice in HB-NC4 low-dose and HB-NC4 high-dose groups was significantly decreased (^☆^ *p* < 0.05 or ^△△^ *p* < 0.01).

We demonstrated here that after intra-articular injection of NC4, it was not retained in the joint cartilage long enough to interact with chondrocytes. Therefore, the therapeutic effect of the NC4 group was not as good as that of the HB-NC4 group. With its prolonged activity, after 12 weeks of continuous intra-articular injection, compared with NC4 and model group, the HB-NC4 fusion protein significantly lowered C5b-9 content in mouse serum, reduced progression of cartilage loss in mice OA model, and lowered C5b-9 content in cartilage, which showed HB-NC4 did reduce the generation of C5b-9 in OA mice (Zhao et al., 2014). Therefore, we hypothesized that the fusion protein, HB-NC4, may become a complement inhibitor with the ability to solve the limitation by therapeutic proteins’ rapid clearance from the joint space and lack of retention within cartilage targeting abilities. Simultaneously, we recognize that HB-NC4 as a potential therapy for OA is suitable for the initial treatment of OA; as OA progresses, the content of glycosaminoglycans decreases; subsequently, the identifiable glycosaminoglycan targets that heparin enters into the articular cartilage will also be reduced; and the efficacy of HB-NC4 will also be lower (Vincent et al., 2007; Miller et al., 2010).

## Conclusion

The expression and purification of HB-NC4 were reported for the first time. Briefly, this work demonstrates that HB-NC4 can be expressed in *E. coli* and purified with high efficiency. Currently, the anti-complement activity and affinity of HB-NC4 are being further investigated. HB-NC4 has no effect on the original anti-complement activity of NC4; instead, it significantly improves the targeting of NC4 to articular cartilage. Further, this study strongly suggests that HB-NC4 inhibits the formation of C5b-9 in OA mice and can effectively relieve inflammation and articular cartilage damage in OA mice. Furthermore, the effect of HB-NC4 in the joints is significantly stronger than that of NC4. This strategy of improving the targeting of therapeutic protein drugs in cartilage is greatly promising for the therapy on OA.

## Data Availability

The datasets presented in this study can be found in online repositories. The names of the repository/repositories and accession number(s) can be found in the article/supplementary material.
